# Continuing Professional Development ‐ Medical Imaging

**DOI:** 10.1002/jmrs.757

**Published:** 2024-01-25

Maximise your CPD by reading the following selected article and answer the five questions. Please remember to self‐claim your CPD and retain your supporting evidence. Answers will be available via the QR code and online at www.asmirt.org/news-and-publications/jmrs, as well as published in JMRS — Volume 71, Issue 4 December 2024.

## Medical Imaging — Original Article

### Optimising image quality in intravenous cerebral cone beam computed tomography

Broadley L, Erskine B, Marshall E, Ewert K, Picker B. (2024). *J Med Radiat Sci*. https://doi.org/10.1002/jmrs.735

1. According to evidence cited in this article, what is the permanent neurological complication rate for cerebral digital subtraction angiography (DSA)?
  - 0.5–0.8%
  - 2–5%
  - 5–7%
  - 8–10%
2. In this study, when only the acquisition field of view (FOV) was modified, the use of which acquisition field size was associated with the highest signal‐to‐noise ratio (SNR)?
  - 22 cm
  - 32 cm
  - 42 cm
  - 48 cm
3. In this study, the use of which acquisition field size produces images with the greatest inherent spatial resolution?
  - 22 cm
  - 32 cm
  - 42 cm
  - 48 cm
4. The use of a low kV imaging technique is advantageous for enhancing the Hounsfield Unit (HU) of iodinated materials. What does this result in?
  - Lesser predominance of the photoelectric effect.
  - Greater predominance of the photoelectric effect.
  - Greater predominance of Compton scattering.
  - Greater predominance of Rayleigh scattering.
5. When compared to intravenous cerebral Cone Beam Computed Tomography (IV CBCT) acquired using a 22 cm field size, what does the use of the 42 cm field result in, as found in this study?
  - Better spatial resolution
  - Higher air kerma
  - Higher dose area product (DAP)
  - Lower HU of the iodinated material

### Recommended further reading

1. Gölitz P, Struffert T, Kaschka I, Roessler K, Knossalla F, Doerfler A. Optimized angiographic CT using intravenous contrast injection: a noninvasive imaging option for the follow‐up of coiled aneurysms? *AJNR Am J Neuroradiol* 2014; 35(12): 2341‐7. https://doi.org/10.3174/ajnr.A4039
2. Brenner DJ, Doll R, Goodhead DT, et al. Cancer risks attributable to low doses of ionizing radiation: assessing what we really know. *Proc Natl Acad Sci U S A* 2003; 100(24): 13761‐6. https://doi.org/10.1073/pnas.2235592100

## Answers

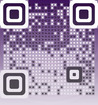

Scan this QR code to find the answers, or visit www.asmirt.org/news-and-publications/jmrs

